# Effect of occupational exposure to welding fumes and noise on heart rate variability: An exposed-unexposed study on welders and airport workers' population

**DOI:** 10.3389/fpubh.2022.937774

**Published:** 2022-09-28

**Authors:** David Lucas, François Guerrero, Emmanuel Jouve, Sophie Hery, Pascale Capellmann, Jacques Mansourati

**Affiliations:** ^1^Center of Environmental and Occupational Diseases, Department of Occupational Health, Brest Teaching Hospital, Brest, France; ^2^ORPHY Laboratory, Department of Sciences, Occidental Brittany University Brest, Brest, France; ^3^Carsat Bretagne (Regional Agency of Occupational Health), Department of Occupational Prevention, Rennes, France; ^4^Occupational Health Service, Department of Occupational Prevention, Naval Group, Brest, France; ^5^Iroise Occupational Health Service, Department of Occupational Prevention 22 Rue de Kervezennec, Brest, France; ^6^Cardiology Unit, Department of Cardiovascular Diseases, Brest Teaching Hospital, Brest, France

**Keywords:** welding fumes, heart rate variability, noise, occupational health, autonomic nervous system, occupational toxicology

## Abstract

**Introduction:**

Welding fumes (WF) are a complex mixture of gas and particles. Action of occupational exposure to WF on cardiovascular system has been recently studied as for noise.

**Research question:**

The main objectives of our study are therefore to evaluate the impact of exposure to WF, noise, and combined WF and noise on autonomic nervous system as assessed by heart rate variability (HRV).

**Methods:**

The study groups were 16 welders and eight airport workers (as a control group). All the participants underwent ambulatory electrocardiogram, personal WF, and noise exposure monitoring, respectively, with dust track and calibrated noise dosimeter during workday. Atmospheric environmental assessments at workplaces have been also performed. HRV parameters were summarized for all the workday and hourly. Correlation tests were used to examine relation between HRV parameters and levels of noise exposure in the two population. Analysis of covariance (ANCOVA) was used for mean of each HRV parameters.

**Results:**

For HRV parameters, we found significant higher levels for mean range of high frequency (HF), standard deviation of normal-to-normal R-R interval (SDNN), and root mean square of successive heartbeat interval difference (RMSSD) in welders which suggested an imbalance between sympathetic and parasympathetic nervous system in this population. For relation between noise and HRV parameters, we noted that levels of low frequency (LF), HF, and SDNN were significantly correlated with mean noise levels for welders (respectively, r = 0.62, r = 0.357, r = 0.48), not in control group. Using ANCOVA, we found that working as a welder significantly increases mean of HF (*p* = 0.01) and RMSSD (*p* = 0.02) and decreases in LF/HF (*p* = 0.008). Indeed, the interaction between exposure to WF and mean noise levels for HF (*p* = 0.005), LF/HF (*p* = 0.01), and RMSSD (*p* = 0.007) was significant.

**Conclusion:**

This study shows an impact of WF and noise on ANS balance. One hypothesis is WF exposure could increase sensibility to noise exposure on autonomic nervous system or there is a synergic effect.

## Introduction

A significant and positive correlation between exposure to WF and the risk of cardiovascular diseases (CVD), especially ischemic heart diseases, has been previously reported (1–3). Workers who carry out welding activities are exposed, through these fumes, to fine particles, ultrafine particles, gases, and metals (4). Two main hypotheses are suggested: (1) a direct impact of WF on the cardiovascular system with a dysregulation of the autonomic nervous system balance and (2) the activation of inflammatory/oxidative stress responses. A significant relationship between exposure to fine particles and both increase in blood pressure and decrease in HRV has been reported (5–7).

Two recent meta-analyses on the impact of noise on cardiovascular system concluded that living or working in an environment with noise exposure is associated with an increased risk of hypertension (OR: 1.62; CI 95% 1.40–1.88), CVD (RR: 1.34; CI 95% 1.15–1.56), and cardiovascular mortality (HR 1.12; CI 95% 1.02–1.24) (8, 9). Pathophysiological mechanisms of long-term effects of noise on cardiovascular system include activation of the autonomic nervous system, which may lead to altered HRV, oxidative stress, and vascular dysfunction (10). Indeed, studies focused on the effects of noise showed both an imbalance of the ANS toward sympathetic activity and an increased risk of CVD (11, 12).

HRV is a non-invasive method to evaluate the balance of sympathetic and parasympathetic branches of the autonomic nervous system (13, 14). Time and frequency domain of HRV have been described by Schaffer recently (15). Furthermore, a retrospective analysis of the data collected from the CAST (Cardiac Arrhythmia Suppression Trial) study showed that the deterioration of HRV 1 year after a myocardial infarction was an independent risk factor for cardiac mortality (16). A decreased HRV has been shown indeed to be a predictor of severe arrhythmia independent of other factors, and other studies have also illustrated that some parameters of HRV are prognostic factors for survival (16–19). It has been suggested that HRV reflects the overall capacity of the body to deal with ongoing demands (20). In this way, HRV may act as a biomarker when considering the influence of occupational and environmental exposures on health-related mechanisms. In regard to the need of biomarkers, HRV has been used not only in cardiovascular diseases but also in psychiatric disorders, epilepsy, and impact of nutrition on health (20–22).

Welders are exposed both to WF and noise at work. However, we are not aware of studies reporting the combined effects of WF and noise in this population. The main objectives of our study are therefore to evaluate the impact of exposure to WF, noise, and combined WF and noise on autonomic nervous system as assessed by HRV.

## Methods

All participants were informed about the potential risks and discomforts associated with the study and gave their written informed consent prior to study enrollment. The protocol was approved by the Ethical Committee of the University Hospital of Brest and performed in accordance with the guidelines of the Helsinki Declaration for human research.

This prospective study included two groups of workers: (1) ship repairing welders were exposed to noise and WF and (2) airport workers were exposed to noise only. We previously showed that airport workers had same cardiac status, educational level, and shift work conditions than welders (23). Airport workers included in the study were assigned to luggage handling and used electric vehicles on the tarmac. They work in a regional airport without a high-level traffic and never stand behind airplanes when engines are running. The primary endpoint is the difference in HRV between these two populations. Inclusion criteria were age > 18 years, national insurance affiliation, welding or airport agent activities, and signed informed consent. Subjects were excluded if minors, medication intake with known effect on endothelial function or coagulation, history of ischemic, thrombotic, or chronic inflammatory disease, pregnancy, and refusal to participate in the study.

### Investigation and follow-up of employees

The study population was followed by the occupational health service. A special information session was provided by an occupational physician before inclusion. After acceptance, a complete medical and professional history, and a medical examination with blood pressure measurement were conducted by an occupational physician.

### Exposure assessment

WF exposure assessment was conducted by trained hygienists. Air samples were taken individually at work in a boat and at the airport (filter holder were placed on worker's shoulders) and environmental samples in different locations of the boat and the airport. Specific methods for air sampling are French-validated methods including Metropol M-274 for WF, M-43 for hexavalent chromium, and M-122 for other metals (24–26). For hexavalent chromium, the inhalable fraction sample of the aerosol is carried out on a quartz fiber filter impregnated with a solution of sodium carbonate/magnesium sulfate. For welding fumes, sampling was made with a specific membrane (pore diameter 5 μm) and analysis by gravimetry (24, 25). Finally, a filter with quartz fiber for sampling and chemical analysis was used for metals (26). Atmospheric samples were performed for airport workers using specific methods including Metropol HAP M-332 and M-188. A quartz fiber filter for air sampling and chromatography for analysis were used (27, 28). Samples were analyzed in a national laboratory affiliated to the regional insurance CARSAT (Caisse d'Assurance Retraite et de la Santé au Travail). A chemical engineer from CARSAT supervised all atmospheric samplings. Work activities during which different samples were taken represented both usual and conventional working conditions.

For noise exposure evaluation, employees wore a calibrated noise dosimeter (Bruel and Kjaer 4418 ATEX, Naerum, Denmark) on their shoulders. The dosimeter allowed noise level measurements between 20 and 140 dB(A) every second. The measurements were A-weighted dB(A). All measurements were performed by an experimented occupational health technician. Data were analyzed with specialized software B&K 2245 Work Noise Partner. This process was in accordance with the AFNOR standard in ISO 9612 (May 2009). To assess noise exposure, we used two indicators: LAEq [average level of sound exposure per hour in dB(A)] and LPC [peak sound pressure level in dB(A)]. Workers from the two groups wore same type of ears' sound protection.

### Measurement of heart rate variability

We used an ECG Holter monitor (ELA Medical SYNESCOPE MultiChannel-MultiDay Version 3.10 Milan, Italy). Workers wore a standard five lead ECG Holter monitor at work. HRV measurement was performed during all workday, began in the morning just before the beginning of the workday. For testing and references' values, the monitoring began by 10 min without physical activity. Digital recordings were analyzed by a trained cardiologist in the Cardiology Department of the University Hospital of Brest. The ECG recordings were made from three orthogonal leads of Frank. The SyneScope ECG Holter analysis software processed the data collected by the PCMCIA Sorin CRM Flash Sorin digital ECG recorders. An automatic and then manual reading of the R-R intervals was performed to ensure the correct identification and classification of each QRS complex as well as the removal of artifacts. Following the recommendations of the Task Force of the European Society of Cardiology and the North American Society of Pacing and Electrophysiology, the presence of more than 20% of artifacts resulted in a rejection of the analysis (29). The sampling frequency of the ECG signal was 20 0Hz. SyneScope calculated the time domain parameters recommended by the Task Force: the RR-maximum and minimum interval, the Heart Rate, SDNN, SDANN, RMSSD, and percent of differences of adjacent RR intervals >50 msec PNN50 (24). The power spectrum density was calculated for the following frequency bands: low (low frequency, LF): 0.05–0.15 Hz and high (high frequency, HF): 0.15–0.35 Hz.

### Statistical analysis

We studied the variation in HRV per 1-h time period as a function of the level of noise exposure. Due to the non-linear nature of sound exposure, responses of the ANS are not homogeneous. Therefore, we carried out an analysis by time segments. We measured the main HRV criteria on each of these fragments independently of each other. Statistical analysis was performed with R software (R Foundation for Statistical Computing, Vienna, Austria). The Shapiro–Wilk test was used to test normal distribution of data. When variables were not normally distributed, non-parametric test (Mann–Whitney U-test) was used. The *t*-test was applied in case of normal distribution. The quantitative variables were described using the usual position and dispersion statistics. The differences for HRV among the two populations were initially assessed by a one-way analysis of variance (ANOVA) followed by an analysis of Covariance (ANCOVA) to statistically control the effect of covariates. The alpha risk was set at 5% for all analyses. Values in tables are expressed as mean ± standard deviation (SD).

## Results

### Population characteristics

From the initial population of 17 welders and 10 airport workers, 16 welders and eight airport workers were finally included with available HRV and noise exposure measurements during a workday. The level of ECG artifacts was >20% in three subjects (one welder and two airport workers) who were excluded from the study. The mean age of welders' population was 42.4 years, they had 18.2 years of experience, and 67% were smokers. For blood pressure, in subject population of welders, blood pressure was, respectively, 71 mm Hg for diastolic and 125 mm Hg for systolic. All subjects were males without differences in smoking status, age, precedence at workstation, body mass index (BMI), and blood pressure between the two populations. No cardiovascular disease was present in all the included subjects. All population characteristics are summarized in Table 1.

**Table 1:** Population characteristics.

	**Welders (*****n*** = **16)**		**Airport workers (*****n*** = **8)**		***p*-value**
	**Mean**	**SD**	**Mean**	**SD**	
Age^a^ (years)	42.4	(±9.30)	45.5	(±7.58)	0.38
Working years as welders/airport workers^a^	18.2	(±9.52)	9.83	(±6.46)	0.11
BMI^a^	24.6	(±4.41)	28.8	(±6.89)	0.24
BPd^a^	71.0	(±8.05)	77.5	(±4.18)	0.14
BPs^a^	125	(±10.5)	122	(±11.7)	0.61
smockers^b^	4 (67%)		4 (67%)		1

^a^Presented as mean+/- standard deviation.

^b^Presented as number of cases (percentage).

BMI, body mass index.

BPd, blood pressure diastolic in mm Hg.

BPs, blood pressure systolic in mm Hg.

### Welding fume exposure assessment

In Welders' group, 150 individual samples and 32 samples from the working environment were performed during 9 days and 41 employees were assessed. We found particles ranging from 10 to 100 microns and respirable particles (< 10 microns). The respirable dust concentrations at the workplace ranged from 0.4 mg/m^3^ to 25.4 mg/m^3^. The mean concentration was 4.5 mg/m^3^, and the median was 1.8 mg/m^3^. We also evaluated the concentrations of respirable dust in the working environment in the ballasts and in the engine room where welders worked. Concentrations of respirable dust ranged from 0.2 mg/m^3^ to 7.5 mg/m^3^. The mean concentration was 1.6 mg/m^3^, while the median was 1 mg/m^3^. The results were below the recommended limit values for French professionals (30). The results are summarized in Table 2. The atmospheric assessment of airport agents required 1 day of intervention, nine employees from two different stations, and 12 individual samples. The concentrations of inhalable particles were all below the limits of quantification. We also measured atmospheric levels of polycyclic aromatic hydrocarbon and concentrations of benzene and benzo(a) pyrene, and they also were below the limit of quantification. The results are summarized in Table 3.

**Table 2:** Results of welding fume exposure assessment.

	**Welders**			**Environmental**			
	**Respirable dust mg/m3**	**CrVI μg/m^3^**	**Mn mg/m3**	**Respirable dust mg/m3**	**CrVI μg/m^3^**	**Mn mg/m3**	**Total**
	62	27	61	20	12	20	**182**
Mean	4.5	2.73	0.13	1.6	0.73	0.06	
Min	0.4	0.00	0.00	0.2	0.00	0.01	
Max	25.4	27.51	1.20	7.5	3.78	0.44	
Median	1.8	1.41	0.05	1.0	0.03	0.03	
8-TWA-	18.00 %	141	2.5	10.00 %	30	1.5	
TLV of
Welding
fumes

Min, minimum.

Max, maximum.

Cr VI, hexavalent chromium.

Mn, manganese.

**Table 3:** Results of exposure assessment of airport workers and comparison to TLV 8h.

	**Airplane assistance**			**Loading unloading luggages**		
	**Results**	**TLV 8h**	**% of TELV**	**Results in mg/m3**	**TLV 8h**	**% of TLV**
Elemental Carbon	< 0.009	0,1	9 %	< 0.006 mg/m3	0.1	6
Hydrocarbon C6-C12 ^a^	< LOQ	1000	-	< LOQ	1000	-
Hydrocarbon C9-C12 ^a^	< LOQ	150	-	< LOQ	150	-
Benzene ^a^	< LOQ	3.25	-	< LOQ	3.25	-
Benzo(a)pyrene ^b^	< LOQ	150	-	< LOQ	150	-
Naphtalene ^b^	129.2	50000000	0.002%	202,1	50000000	0.004

^a^in mg/m3.

^b^in ng/ m3.

TLV 8h, threshold value levels on 8 h of work.

LOQ, limit of quantification.

### Noise exposure assessment

For noise exposure measurement, six participants of each group wore a dosimeter for a total of 76 acoustic sequences. The average workday noise exposure for welders was 83.1 and 83.6 dB(A) in the airport. We did not find differences for the average level of sound exposure per hour. For peak sound pressure level per hour, mean values, respectively, for airport workers and welders are 128 and 124 dB(A). However, LPC was statistically significantly higher (*p* = 0.019) for airport workers than for welders not mean value for a workday.

The results are summarized in Table 4.

**Table 4:** Noise exposure assessment results.

	**Welders' exposure time** ***N*** = **37**		**Airport workers' exposure time** ***N*** = **39**		
	**Mean**	**SD**	**Mean**	**SD**	***p*-value**
LAEQ	83.6	±5.89	83.1	±7.48	0.76
LPC	124	±5.13	128	±6.47	0.019*

LAEQ, average level of sound exposure per hour in A-weighted dB(A).

LPC, Peak sound pressure level per hour in A-weighted dB(A).

^*^p-value < 0.05.

### Holter analyses

#### HRV parameters on 8 h

Considering the whole workday, no statistical difference was found in heart rhythm, conduction. or repolarization disorders between welders and airport workers. The maximum and minimum RR interval did not differ between the two groups (*p* = 0.36 and *p* = 0.19). The mean heart rate during workday did not differ between the two groups (94.5 ± 10.1 vs. and 91.3 ± 5.44 bpm, *p* = 0.24).

HRV measurements and comparison between mean values on 8 h in time and frequency domains are presented in Table 5. We found significantly higher values of RMSSD (*p* = 0.04) and SDNN (*p* = 0.05) in welders.

**Table 5:** Differences in time and frequency domains of HRV values between welders and airport workers.

	**Welders' exposure time** ***N*** = **37**		**Airport workers' exposure time** ***N*** = **39**		***t*–value**	***p*–value**
	**Mean**	**SD**	**Mean**	**SD**		
LF	928.8	± 481	852.56	± 490	−0.57	0.07
HF	198.16	± 160	88.38	±48	−4.15	0.02*
LF/HF	6.6	± 3.4	9.6	±4.5	3.38	0.33
SDNN	57.46	± 13.4	49.15	±16	2.37	0.05*
RMSSD	27.76	± 16.7	18.28	±5.4	−3.81	0.04*

SD, standard deviation.

p. p–value.

The p– and t–values were derived from Mann–Whitney U–test.

^*^p < 0.05 difference is significant at 0.05 levels.

### Relation between HRV parameters and noise exposure

We obtained 76 1-h time segments evaluating daily exposure and peak sound pressure (39 for airport workers and 37 for welders).

First, the results of an univariate linear regression are described in Tables 6, 7.

**Table 6:** Correlation between HRV value per hour and mean noise measurements per hour by group of workers with Pearson's correlation test.

	**Welders' exposure time *N* = 39**	**Airport workers ‘exposure time *N* = 37**
	**Pearson's correlation coefficient**	**Pearson's correlation test**
LF	r = 0.62*	r = 0.12
HF	r = 0.357*	r = 0.073
LF/HF	r =-0.06	r = 0.0902
SDNN	r = 0.48*	r = 0.03205
RMSSD	r = 0.238	r = 0.1282

r, Pearson's correlation coefficient.

^*^p < 0.05 relation is significant at 0.05 levels.

**Table 7:** Correlation between HRV value per hour and peak noise measurements per hour by group of workers with Pearson's correlation test.

	**Welders' exposure time** ***N*= 39**	**Airport workers exposure time** ***N* = 37**
	**Pearson's correlation coefficient**	**Pearson's correlation test**
LF	r = 0.141	r = 0.04
HF	r = 0.297	r =-0.056
LF/HF	r = −0.300	r = 0.32*
SDNN	r = 0.28	r = 0.062
RMSSD	r = 0.25	r = −0.015

r, Pearson's correlation coefficient.

^*^p < 0.05 relation is significant at 0.05 levels.

For welders' population, we observed a statistically significant positive correlation between 1-h time value of HRV and mean noise values for LF (r = 0.62, *p* < 0.05), HF (r = 0.357, *p* < 0.05), and SDNN (r = 0.48, *p* < 0.05). No significant correlation with peak of noise was found.

In airport workers' population, none of the HRV parameters were significantly correlated with the level of noise exposure.

Second, we performed an ANCOVA on all HRV parameters as dependent variables, time at workplace, workplace, and mean sound levels as covariates. The best model does not include time at workplace. The results summarized in the Table showed that workplace exerts a statistically significant influence for all HRV parameters except LF and SDNN while mean noise had effect for LF, HF, and SDNN. A statistically significant workplace ^*^ mean noise interaction was detected for RMSSD, HF, and LF/HF (Table 8).

**Table 8:** ANCOVA results for mean sound level and workplace on HRV parameters.

		**LF**	**HF**	**LF/HF**	**RMSSD**	**SDNN**
R^2^		0.291	0.232	0.115	0.197	0.194
F		9.257	6.793	2.919	5.533	5.431
P		0.000	0.003	0.064	0.007	0.008
Mean sound level	F	18.450	4.628	0.185	2.050	6.946
	p	< 0.0001*	0.037*	0.669	0.159	0.011*
Workplace	F	0.129	7.063	5.831	7.668	2.511
	p	0.721	0.011*	0.020*	0.008*	0.120
Mean sound level* Workplace	F	Not included	8.689	6.207	7.945	2.784
	p		0.005*	0.016*	0.007*	0.102

R^2^, determination coefficient.

F, Fisher's coefficient.

P, p–value.

*p < 0.05 relation is significant at 0.05 levels.

Altogether, our data indicate that HF is increased in welders but decreased in airport workers, whereas LF is unchanged in both groups. Indeed, the results showed an increased RMSSD and SDNN with a higher level of noise exposure in welders but not airport workers, which is associated with higher HF and decreased LF/HF in welders while lower HF and increased LF/HF in airport workers. The interaction between mean sound levels and working as welders have a significant increase on RMSSD and HF and decrease for LF/HF levels.

## Discussion

In our study population, HRV analysis shows that mean range of HF, SDNN, and RMMSD in welders was significantly higher than in airport workers. We found that workplace had a significant effect on all, except LF, domains of HRV, and interaction values with mean noise levels for and HF, LF/HF, and RMMSD. Indeed, only for welders' group, HRV measurements LF, HF, and SDNN increased significantly with mean noise values.

For WF, impact and timing of HRV variation due to exposure to these fumes have been previously studied. The results of monitoring in 36 boilermakers showed a short-term decline in hourly SDNN index in the first hours after exposure to welding fumes, followed by a plateau and a second period of decline in the 9–10 h after cessation of the works (31). Another team included 66 male boilermakers with HRV measurements on welding and non-welding days. The differences of HRV measurements are significantly different between pre- and post-shift on welding days but not significant on non-welding days. For each 1mg/m^3^ of PM 2.5 exposure level, there was a 17% decrease in PNN10, 13% in PNN20, and 55% in HF range. There was also a non-significant decrease in SDNN and RMMSD (32). Variations during the night monitoring are more linked to a short-term effect, a few hours after exposure. This was confirmed by another study in which welders have been monitored during 22 h with a circadian variation of HRV over both working days and non-working days. The results showed lower HRV and a change in the circadian pattern on working days (33). In our study, increasing levels of nickel, chromium, and manganese between Monday morning and Friday evening in welders' urinary samples confirmed exposures to WF. WF have an impact on 8-h levels for HF, SDNN, and RMSSD but also for HF, LH/HF, SDNN, and RMSSD in 1-h samples.

Welders with decreased LF/HF ratio should have higher risk of mortality due to cardiovascular diseases. Pagani's group and Schaffer and Ginsberg stated that HRV is a non-invasive method to evaluate the balance between sympathetic and parasympathetic branches of the ANS with LF reflecting the sympathetic branch, HF the parasympathetic, and LF/HF the balance between both (14, 15). In a recent review, Hayano and Yuda distinguished long- and short-term analyses of HRV (19). According to these authors, the use of long-term analysis of the HRV for the evaluation of autonomic nervous function should take into account an estimation of physical activity and posture. In our study, welders' HRV was compared with a population with similar physical activity level and during a working day, thus also excluding differences in posture. Therefore, the lower LF/HF ratio on 8 h in welders' population likely reflects a difference in the autonomic function, and it was associated to an increased risk of mortality in studies on cardiovascular disease prognosis (19).

One study on cardiac arrhythmia and WF exposure was recently published by Cavallari. In a population of 72 male welders, a significant association between PM 2.5exposure and the risk of having ventricular ectopy 6 and 7 h after exposure was reported (34). The correlation between ventricular ectopy and increased PM 2.5per unit suggests that healthy workers are prone to arrhythmogenic effects of particles exposure and could be correlated to ANS imbalance.

In literature, correlations between noise exposure and short-term HRV parameters have been previously shown. In the RECORD study, an increase by one dB(A) in Leq was associated with, respectively, a concomitant increase in 0.97%, 2.08, 1.3, and 1.16% of SDNN, LF, HF, and LF/HF ratio, and in another study, the concurrent increases of 5 dB(A) in Leq were associated with increases in HR (1.48%) and LF/HF (4.89%) but decrease in LF (−3.77%) and HF (−8.56%) (12, 35). In our study, significant relation between LF, HF, and SDNN and mean noise levels have only been found for welders.

According to the Babish's noise effect models, under the involvement of the limbal system and hypothalamus, exposure to noise influences the ANS either directly or indirectly through the stress hormones (36). Indeed, chronic stress due to a “fight-or-flight” response could generate increased blood pressure, lipids, or glucose levels and activation of blood coagulation (37, 38). Munzel hypothesizes that this response to chronic noise-stress is characterized by the activation of the sympathetic system and increased levels of catecholamine, cortisol, and angiotensin II (10). In a recent meta-analyses (67 studies) on different types of sound and their relationship with the ECG signals, the authors found a trend toward an increase in blood pressure. Nonetheless, there is no marked trend for LF and HF between studies (increase, stable, and decrease). Only the LF/HF ratio increased compared to noise exposure (39). Our statistical analysis ANCOVA is for an interaction between noise and welding fume exposures on some parameters of HRV including HF, LF/HF, and RMMSD. Indeed, the only significant correlation for HRV parameters (LF, HF, and SDNN) and mean noise exposure, and the elevated 8-h values of RMSSD and SDNN in welders put out the question of joint effects on ANS imbalance.

Moreover, significant interaction between working as welder and mean sound level on decrease in LF/HF and increase for HF strengthened interrogation. Is it due to cumulative or synergic effects? We could hypothesize that noise and WF exposure generated activation of baroreflexes with imbalance of ANS. In his review on the utility of LF in HRV, Reyes Del Paso concluded that HRV spectrum in frequency domains is predominantly determined by vagal activity and that HRV analysis is an estimation of the parasympathetic influences on HR (40). For Goldstein, LF power is associated with baroreflex modulation of autonomic outflows (41). Respiratory sinus arrythmia is one of the major components in short-term HRV and is influenced by non-vagal factors (19, 42).

In case of acute exposure, autonomic nervous system could regulate the influence of the sympathetic tone, but after few minutes for noise and few hours for WF, this capacity is overloaded. The cavallary's study suggests that exposure to WF has a double time effect with a very short-term and short-term impact on the ANS balance (33). The co-exposure of two different environmental factors of activation of baroreflexes through autonomic nervous branches could limit adaptation of this system in time. Other factors could influence HRV in our study: regular physical exercise at work which is known to increase sensitivity of the cardiac baroreflex and to reduce sympathetic branch action; respiratory capacities of workers and time of standing increase LF/HF (29, 43). Indeed, even if the two groups had a generally similar cardiac strain, it cannot be ruled out that the welder group performed much more sustained work than the airport workers during our analysis schedules. Maybe, time of exposure of our included workers is too short for such stimulation.

### Limitations

The small number of subjects included in this study is the main limitation of our results. The design of the study allowed us to analyze 76 1-h noise and ECG measurements. Reducing these sequences to 5 min allowed us to analyze a larger number of them and thus increased the power of the study. This study did not include any women. Therefore, no analysis of gender effect is possible. Another limitation of this study is the lack of information on confounding factors such as temperature, external stimuli as light exposure, time of day, and food consumption (44, 45). Caffeine is a possible stimulator of the sympathetic nervous system. Most of the subjects drank coffee during breaks and breakfast. In addition, we noticed a large difference between our results and published values for both pathological and normal values. Finally, we did not perform echocardiograms to evaluate cardiac structure.

## Conclusion

This study shows an impact of WF and noise on autonomic nervous system balance. It seems that cardiovascular system could react during a short time to noise and WF exposure through baroreflex regulation. After minutes or hours, regulation is overloaded with imbalance of ANS. According to ECG monitoring during working time, significant effect of workplace, and interaction between WF and noise exposures on HRV parameters, we could suggest that WF exposure could increase sensibility to noise exposure on ANS or there is a synergic effect. Further studies are needed to explain pathophysiological pathways and the possible cardiovascular impact of both factors or a potentiating effect of noise.

## Take-home message

Noise and welding fumes' exposure have an impact on heart rate variability and autonomic nervous system. It could explain higher risk of cardiovascular diseases in population exposed to noise and welding fumes.

## Wider context

A possible combined effect of gas and fine particles (welding fumes) and noise on autonomic nervous system need confirmation and open questions on other pathways such as oxidative stress, endothelial dysfunction, and thrombosis when humans are exposed to these compounds.

## Data availability statement

The raw data supporting the conclusions of this article will be made available by the authors, without undue reservation.

## Ethics statement

The studies involving human participants were reviewed and approved by Comité de Protection des Personnes Ouest II ref 2017-AO1829-44. The patients/participants provided their written informed consent to participate in this study.

## Author contributions

FG, JM, and DL: study design. EJ: exposure assessment. PC: noise assessment. DL, SH, and FG: inclusion, clinical examination, and Holter ECG on participants. FG and DL: data analysis. FG, JM, and DL: article writing. EJ, SH, and PC: corrections on article. All authors contributed to the article and approved the submitted version.

## Funding

This study was funded by the Research and Innovation Unit Brest Teaching Hospital Brest, France.

## Conflict of interest

The authors declare that the research was conducted in the absence of any commercial or financial relationships that could be construed as a potential conflict of interest.

## Publisher's note

All claims expressed in this article are solely those of the authors and do not necessarily represent those of their affiliated organizations, or those of the publisher, the editors and the reviewers. Any product that may be evaluated in this article, or claim that may be made by its manufacturer, is not guaranteed or endorsed by the publisher.
